# Bilateral Optic Neuritis Secondary to Nivolumab Therapy: A Case Report

**DOI:** 10.3390/medicina54050082

**Published:** 2018-11-06

**Authors:** Ömer Kartal, Erman Ataş

**Affiliations:** 1 Division of Pediatric Hematology and Oncology, Gülhane Training and Research Hospital, 06300 Ankara, Turkey; 2 Division of Pediatric Oncology, Gülhane Training and Research Hospital, 06300 Ankara, Turkey; eatasdr@gmail.com

**Keywords:** glioblastoma multiforme, nivolumab, optic neuritis

## Abstract

Pediatric glioblastoma multiforme is an uncommon and highly mortal brain cancer. New therapeutic treatments are being intensively investigated by researchers in order to extend the survival of patients. The immune checkpoint inhibitor nivolumab in the treatment of pediatric glioblastoma multiforme is currently under review; it is a human immunoglobulin G4 monoclonal antibody that works against the programmed cell death protein 1 receptor, designed to enhance an immunologic reaction against cancer cells. Herein, we describe the first report of a bilateral optic neuritis induced by nivolumab in a grade 4 glioblastoma multiforme patient.

## 1. Introduction

The current standard treatment for the newly diagnosed pediatric glioblastoma multiforme (GBM) is surgical debulking followed by radiation therapy and chemotherapy. However, a 5-year survival rate is less than 10%, and the median survival is 1–2 years [1]. Therefore, there is an urgent need to develop new therapeutics that can improve survival in GBM.

The immune checkpoint inhibitor nivolumab has been shown to improve antitumour response in a number of different advanced malignancies in different studies. The treatment of pediatric GBM is currently under review. It is a human immunoglobulin G4 monoclonal antibody that works against the programmed cell death protein 1 receptor, and is designed to enhance an immunologic reaction against cancer cells. On the other hand, using immune checkpoint inhibitors may cause an autoimmune phenomenon, including pneumonitis, hepatitis, vitiligo, colitis, hypophysitis, pruritis, arthritis, nephritis, neurologic syndromes (e.g., aseptic meningitis, Guillain-Barre’ syndrome), and autoimmune hemolytic anemia [2]. Herein, we describe the first report of a bilateral optic neuritis induced by nivolumab in an advanced GBM patient. 

## 2. Case Presentation

**Figure 1:**
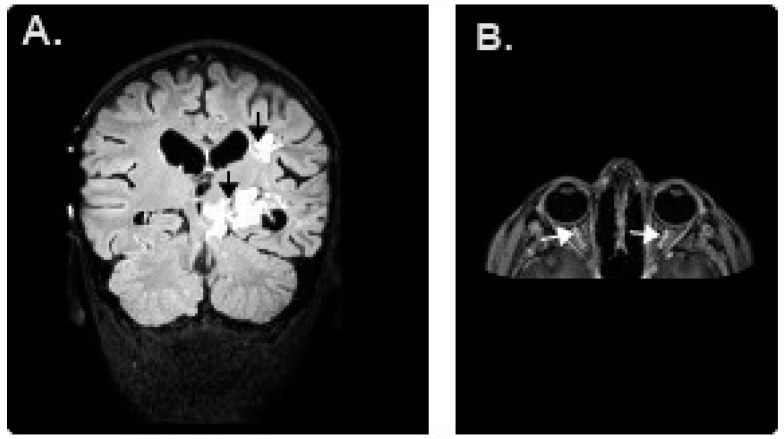
(**A**) Magnetic resonance image (MRI) of the patient’s brain demonstrating a left brain hemisphere and brainstem glioblastoma; (**B)** On T1-weighted sequence, after contrast injection, an enhancement of the bilateral optic nerve compatible with optic neuritis.

The patient was a 9 year old male with a 10-day history of severe headache, vomiting, and numbness in the right arm and foot. Bilateral papilledema was found at the ophthalmic examination. Magnetic resonance imaging (MRI) displayed a mass with homogenous contrast enhancement in the left brain hemisphere and brainstem (Figure 1). On the operation, a subtotal excision was performed. Histopathological examination of the excisional piece revealed a malignant tumor that had anaplasia, marked cellularity, necrotic areas, and a remarkable neoangiogenesis with proliferation of endothelium of the capillaries. The tumor was histopathologically diagnosed as a glioblastoma multiforme. The subtotal excisional surgery was followed by cranial radiotherapy with a total dose of 60 Gy. Then we applied temozolomide (200 mg/m^2^/day peroral for 5 days; every 4 weeks for 10 cycles) and bevacizumab (10 mg/kg IV; every 2 weeks for 6 cycles) plus irinotecan (125 mg/m^2^ IV; every 2 weeks for 6 cycles) as first and second-line treatments. However, in the control magnetic resonance imaging, the tumor showed progression despite these treatments. Therefore, we began to use nivolumab as a third-line treatment.

Nivolumab therapy was started at a dose of 3 mg/kg intravenously every two weeks. Two days after the second dose, the patient was admitted to the hospital with a rapidly progressive decline in visual acuity of the eyes. On ophthalmic examination, the visual acuity of the right eye was counting fingers at 1 m and was very low on his left eye (limited to light perception). At the posterior segment examination, there was an optic disc swelling bilaterally. Other vital findings, such as blood electrolyte levels and neurological examination, were normal. An urgent MRI showed bilateral thickening of the optic nerves suggestive of optic neuritis, with normal intracranial pressure (Figure 1). Bilateral optic neuritis was diagnosed with the combination of clinical features, ophthalmic examination and radiological findings. Bilateral optic neuritis was diagnosed four days after the progressive decline in visual acuity of the eyes and, first we stopped nivolumab therapy and then the patient began pulse dose steroids; he received intravenous corticosteroids (1 g/day) for 5 days, which resulted in a progressive improvement in visual acuity. After a week, the vision improved to 20/20 in both eyes and he did not need any additional treatment at the next follow-up.

## 3. Discussion

As a treatment option, nivolumab and other immune checkpoint inhibitors are beginning to be preferred in daily clinical practice by researchers. Therefore, immune-related adverse events have significantly increased, however, the adverse events are reported to be less undoubtedly in clinical studies. For example, pneumonitis, type I diabetes mellitus, pruritis, encephalitis, myasthenia gravis, hepatitis, thyroiditis, colitis and other autoimmune diseases can emerge in any part of the body [2,3]. To the best of the authors’ knowledge, this is the first case of nivolumab-associated optic neuritis confirmed by the combination of clinical features, ophthalmic examination, and radiological findings.

In one case reported by de Velasco and colleagues, nivolumab was associated with uveitis after cycle 28 (10 mg/kg, every 3 weeks) and in another case reported by Karlin and colleagues, the patient developed bilateral uveitis after cycle 10 (3 mg/kg, every 2 weeks) [4,5]. In contrast to these cases, the uveitis was not seen in our patient, however, we encountered bilateral optic neuritis after cycle 2 (3 mg/kg, every 2 weeks). These cases show that, immun ophthalmic disorders could be seen at a considerably lower cumulative dose of the drug.

According to some clinical studies, a pulse dose of steroids can be used for optic neuritis [6]. In the study that was made by Beck and colleagues, the response to the IV steroid administration was more rapid than after placebo or oral steroids on the 15th day [7]. The rate of return of visual acuity to normal was higher in the IV steroid group than in the placebo group at six months; however, there are not any significant differences between the oral steroid group and the placebo group [7]. Additionally, the benefit of long-term use of steroids is unknown. Jayakody and colleagues found that, there are no differences in outcome between a shorter (two weeks) and longer (more than two weeks) course of steroids in children with optic neuritis [8]. In our case, immediately after the diagnosis, we started treatment by giving of 30 mg/kg per day IV methylprednisolone, maximum 1 g daily, for 5 days. Because of the patient’s good response to the therapy, we did not prolong the steroid treatment. 

In the pediatric period, bilateral optic neuritis is more common in the younger age-group (<10) as compared to the older age-group (≥10). Waldman and colleagues found that optic neuritis is correlated with age [9]. For every 1-year increase in age, the rate of unilateral optic neuritis (compared with bilateral involvement) is increased by 25% [9]. In accordance with this study, our case is also 9 years old and optic neuritis developed bilaterally.

Clinical presentation of optic neuritis may be dramatic, however, the visual outcome of optic neuritis in children, especially in younger children, is fairly good. Wilejto and colleagues evaluated 36 cases with optic neuritis in Canada and found that the visual acuity of 39 of 47 eyes (83%) recovered to ≥20/40 after 2.4 years [10]. Mizota and colleagues evaluated 41 cases and 54 of 61 eyes (95%) recovered to 20/20 or better after a mean follow-up of 10.7 years [11]. In our case, after a week, the vision improved to 20/20 in both eyes. Most likely, this rapid improvement is associated with beginning the pulse steroid treatment immediately.

Probably, nivolumab is responsible for the optic neuritis seen in this patient because of the short duration of time between nivolumab initiation and decrease in visual acuity in this case. Nivolumab, a human immunoglobulin G4 monoclonal antibody against programmed cell death protein 1 receptor, blocks T cell inhibition and stimulates the immunologic response towards cancer cells, however, it may also reduce the self-tolerance of the immune system and may cause an autoimmune phenomenon. Presumably, because of this mechanism the optic neuritis developed bilaterally in our case [12,13].

## 4. Conclusions

Bilateral optic neuritis as an adverse event of nivolumab therapy has not been reported yet. It is the first known report of bilateral optic neuritis associated with nivolumab in childhood. Nivolumab may stimulate many unknown autoimmune disorders and these may become possible limiting factors in the clinical usage of the drug. Further work is also needed to determine the association between nivolumab and optic neuritis.
